# The Research of Toxicity and Sensitization Potential of PEGylated Silver and Gold Nanomaterials

**DOI:** 10.3390/toxics9120355

**Published:** 2021-12-16

**Authors:** Dong-Han Lee, Seo-Yoon Choi, Ki-Kyung Jung, Jun-Young Yang, Ja-young Jeong, Jae-Ho Oh, Sung-Hyun Kim, Jin-Hee Lee

**Affiliations:** Division of Toxicological Research, National Institute of Food and Drug Safety Evaluation, Ministry of Food and Drug Safety, Cheongju 28159, Korea; donghan04@korea.kr (D.-H.L.); sugar0819@korea.kr (S.-Y.C.); kikyung@korea.kr (K.-K.J.); yangjy@korea.kr (J.-Y.Y.); 0jjy@korea.kr (J.-y.J.); chopin68@korea.kr (J.-H.O.)

**Keywords:** skin sensitization, immunogenicity, alternative test, nanomaterials, polyethylene glycol, PEGylation

## Abstract

Polyethylene glycol (PEG) is a polymer used for surface modification of important substances in the modern pharmaceutical industry and biopharmaceutical fields. Despite the many benefits of PEGylation, there is also the possibility that the application and exposure of the substance may cause adverse effects in the body, such as an immune response. Therefore, we aimed to evaluate the sensitization responses that could be induced through the intercomparison of nanomaterials of the PEG-coated group with the original group. We selected gold/silver nanomaterials (NMs) for original group and PEGylated silver/gold NMs in this study. First, we measured the physicochemical properties of the four NMs, such as size and zeta potential under various conditions. Additionally, we performed the test of the NM’s sensitization potential using the KeratinoSens™ assay for in vitro test method and the LLNA: 5-bromo-2-deoxyuridine (BrdU)-FCM for in vivo test method. The results showed that PEGylated-NMs did not lead to skin sensitization according to OECD TG 442 (alternative test for skin sensitization). In addition, gold nanomaterial showed that cytotoxicity of PEGylated-AuNMs was lower than AuNMs. These results suggest the possibility that PEG coating does not induce an immune response in the skin tissue and can lower the cytotoxicity of nanomaterials.

## 1. Introduction

Polyethylene glycol (PEG) is composed of repeating ethylene glycol units and, when it is attached to a polypeptide or another molecule, the phenomenon is referred to as “PEGylation” [1]. PEGylation technique is commonly used in pharmaceuticals, performing an important role in nanoparticle (NP) stabilization [2]. NP is used to transport genes and drugs to target cells and tissues, and PEG prevents the NPs from opsonization, aggregation, and phagocytosis by altering NP’s size and solubility [2]. Therefore, PEGylation protects the content of the delivery carrier from being degraded by proteolytic enzymes and swiftly eliminated by the kidneys, and upregulates the circulation time to increase the delivery efficiency [1,2]. With regard to the abovementioned benefits which overcome the biological limitations, PEGylated NPs have been applied to treatment of various diseases such as cancer, pulmonary diseases, and hepatocellular carcinoma [3,4,5,6].

Substantially, PEGylation of NPs is utilized for messenger ribonucleic acid (mRNA) vaccines of Coronavirus disease 2019 (COVID-19) [7]. The COVID-19 vaccine manufactured by Pfizer and BioN-Tech delivers mRNA, packaged with a PEGylated lipid nanoparticle (LNP) to improve the effectiveness of a vaccine [7]. Anaphylaxis has rarely been reported after vaccination, however mRNA vaccines containing PEG are known to sometimes trigger anaphylaxis [7,8]. The reason for vaccine-related anaphylaxis is still unclear, but PEG is a one of the candidates regarding the causes [8]. The allergic reaction to PEG is uncommon, but it is fatal and can be easily exposed to humans. Thus, concerns about PEG are increasing, and the importance of the safety assessment of PEG is also being emphasized. Therfore, in this study, we aimed to analyze the skin sensitization by the PEGylated NPs based on the Organisation for Economic Cooperation and Development (OECD) Test Guideline (TG) of skin sensitization.

OECD TG 442 (alternative test for skin sensitization) provides a skin sensitization testing method according to dendritic cell activation by adverse outcome pathway (AOP). Through four key events in AOP, skin sensitization could be assessed by in chemico, in vitro, and in vivo test methods [9]. The key events 1 to 3 are in chemico and in vitro assays; in chemico direct peptide reactivity assay (DPRA), in vitro antioxidant responsive element (ARE)-Nuclear factor erythroid 2 related factor 2 (Nrf2) luciferase assay using KeratinoSens™ (Givaudan, Switzerland), and in vitro human cell-line activation test (h-CLAT) using THP-1 [10,11,12,13]. Throughout these experiments, we can verify “haptenation” (key event 1), “induction of inflammatory response” (key event 2), and “activation of monocytes and dendritic cells” (key event 3) [9]. Lastly, the fourth key event is lymph node activation assay (LLNA) to confirm the lymph nodes activation, which is evaluated by enzyme-linked immunosorbent assay (ELISA) and flow cytometry (FCM) [9,14].

Recently, many studies have tried to report the toxicological effect of nanomaterials (NMs) as the NPs-related concern has been elevated. Additionally, application of NM’s surface modification in the biomedical field holds a critical position [15]. However, there is little research investigating the sensitization of PEGylated NPs. Therefore, in this study, we assessed the potential of skin immunogenicity which can be induced by PEGylated-NMs using PEG-coated gold and silver NMs.

## 2. Materials and Methods

### 2.1. Test Nanomaterials

In this study, silver (Ag)/gold (Au) nanomaterials (dispersion) were used for the original group and the PEGylated group according to the attachment of surface functional groups, respectively. Four types of nanomaterials were purchased from Sigma-Aldrich (St. Louis, MO, USA): AgNMs (#730785), PEGylated AgNMs (#796301), AuNMs (#753610), and PEGylated AuNMs (#NCXAUXU30). To profile the hydrodynamic size and zeta potential of four NMs, we used Zetasizer-Nano ZS instrument (Malvern Instruments, Malvern, UK) in different working solutions: PBS (Life Technologies, Grand Island, NY, USA), Dulbecco’s Modified Eagle’s Medium (DMEM) (Life Technologies, San Diego, CA, USA) with 1% heat-inactivated fetal bovine serum (FBS) (Life Technologies), and N,N-Dimethylformamide (DMF) (Sigma) solution with 3% heat-inactivated (60 °C) BALB/C mouse serum. Endotoxin of test NMs was determined by the Endpoint Chromogenic Limulus Amoebocyte Lysate assay kit (Cambrex, Walkersville, MD, USA).

### 2.2. Preparation of NMs Suspensions

We prepared the suspensions of AgNMs, PEG-AgNMs, AuNMs, and PEG-AuNMs for KeratinoSens™ assay by following a slightly modified method from the previous literature [16]. To disperse the NMs, the NMs stock solutions were mixed with PBS solution and sonicated at 40 kHz with 100 W output power for 10 min in a bath sonicator (Saehan-Sonic, Seoul, Korea). Then, the four dispersed NMs solutions were diluted in the fresh DMEM medium containing 1% FBS to make different concentrations of working solutions (0.98–2000 µM). After NMs stock was put with a DMEM medium, it was sonicated for an additional 10 min from the time the aggregation triggered. In LLNA: BrdU-FCM assay, mouse serum was used as a NM’s dispersant [17]. The 3% mouse serum of the final volume was added to the NMs solution and further dispersed for 15 min. Finally, the stock was added to a DMF, being dispersed for an additional 15 min.

### 2.3. Cell Culture

The ARE-element KeratinoSens™ cell, a transgenic human keratinocyte, possesses a stable insertion of the Luciferase reporter gene and is provided from Givaudan Suisse SA (Vernier, Switzerland). KeratinoSens™ were cultured in complete DMEM medium with 10% FBS and 0.5 mg/mL Geneticin (CASRN. 108321-42-2, Sigma, St. Louis, MO, USA). Every 3–4 days, we sub-cultured the cells at 80% confluence for a maximum of 25 passage numbers. The 200 μL of stabilized cells were placed into wells of a 96-well culture plate at a density of 1 × 10^4^ cells/well. Thereafter, the cells were cultured at 37 °C in 5% CO_2_ incubator (overnight).

### 2.4. NMs Suspension Treatments and KeratinoSens™ Assay Methods

The treatment process of NMs was exactly the same between naked NMs (AgNMs and AuNMs) and PEGylated NMs (PEG-AgNMs and PEG-AuNMs). To treat NMs, KeratinoSens™ cells were grown to 80% confluence. The cells were washed using pre-warmed Dulbecco’s phosphate-buffered saline (DPBS) (Life Technologies). After washing, we vehicle controlled and dispersed each NMs suspension (concentration: 0.98–2000 µM) were immediately treated then incubated for 48 h. The trans-cinnamic aldehyde (Sigma) as a positive control was tested in the same method (concentration: 4–64 µM). Additionally, to correct the measured values, a blank control in which no cells were present was provided for each test.

The cell viability was evaluated by the thiazolyl blue tetrazolium bromide (3-(4,5-dimethylthiazo-2-yl)-2,5-diphenyl-tetrazolium bromide (MTT) assay reduction test (CAT #G358B; Promega, Madison, WI, USA). The test method was performed according to the protocol provided by the kit manufacturer. Briefly, a mixture of cell medium and cell proliferation assay kit dye solution was dispensed into each well and incubated at 37 °C for 4 h. After that, stop solution was dispensed per well, pipetted 20 times, incubated at 4 °C for 1 h, and then pipetted 20 times. To exclude color-interference of NMs present in cells or wells, the fully colored supernatant was transferred to a transparent 96-well plate with a pipette and absorbance was measured at 570 nm with a multi-microplate reader (Synergy HTX, BioTek, Seoul, Korea). The cell viability (%) can be calculated based on the optical density (OD) value of the vehicle control (VEH) and blank control.

(1) $$\mathrm{Viability}\;\left(\%\right)=\frac{\mathrm{sample}\;\mathrm{OD}\;-\;\mathrm{blank}\;\mathrm{OD}}{\mathrm{VEH}\;\mathrm{OD}\;-\;\mathrm{blank}\;\mathrm{OD}}\times 100$$

Luciferase activity of tested NMs was judged using the One-Glo™ Luciferase assay kit (CAT #E606A; Promega). The test method was performed according to the protocol provided by the kit manufacturer. Briefly, a mixture of substrate + buffer and phosphate-buffered saline (PBS) was dispensed into each well and incubated at room temperature for 5 min (shaking). After that, luminescence intensity of each sample was obtained using a multi-microplate reader (BioTek). The luciferase induction of four NMs were calculated based on the luminescence values of the VEH control and blank control.

(2) $$\mathrm{Fold}\;\mathrm{induction}=\frac{\mathrm{sample}\;\mathrm{luminescence}-\mathrm{blank}\;\mathrm{luminescence}}{\mathrm{VEH}\;\mathrm{luminescence}-\mathrm{blank}\;\mathrm{luminescence}}$$

### 2.5. Animals

This study was approved by the Ministry of Food and Drug Safety (MFDS) Korea Institutional Animal Care and Use Committee (IACUC) (2021, Approval NO. MFDS-21-017). As validation studies on LLNA: BrdU-FCM have been conducted extensively on BALB/c mice, BALB/c mice were considered as the most appropriate species [18]. The specific pathogen free, female BALB/c mice (seven weeks old) were purchased from Koatech (Pyeongtack, Gyeonggi-do, Korea). The mice were maintained at an animal facility in the MFDS Korea and acclimated for at least five days before experiments. The animals were housed at a temperature of 22 ± 3 °C and a relative humidity of 30–70%. The room was lit with artificial light for 12 h/day. The animals were free to access solid diets and sterilized drinking water.

### 2.6. NMs Treatments and LLNA: BrdU-FCM Assay Methods

The LLNA: BrdU test methods using flow cytometry was performed as previously described in a paper by Han et al. [19] and recently revised OECD TG 442B [18]. On days 1, 2, and 3, the 25 µL of the test materials, vehicle control (DMF contained 3% serum), and the positive control were spread on the back of each ear daily. The positive control was 25% hexyl cinnamic aldehyde in acetone: olive oil (4:1, *v*/*v*). All the test materials were made by the same method freshly before every administration. On day 5, animals were intraperitoneally injected with 100 µL of BrdU mixture (20 mg/mL) in PBS. On day 6, mice were treated, and the auricular-lymph nodes were collected. Lymph nodes were mashed using a cell scraper (SPL, Pocheon, Gyeonggi-do, Korea) to prepare the lymph node cells (LNCs). LNCs were stained with trypan-blue (Sigma) and counted using a hemocytometer. LNCs (1.5 × 10^6^ cells/mL) were prepared using a commercially FITC BrdU Flow Kit (BD Biosciences, San Jose, CA, USA). LNCs were analyzed using BD FACS Calibur™ flow cytometry (BD Biosciences). Stimulation index (SI) values for each NM were calculated using the formula described in the OECD test guideline (TG 442B). If the SI values were above 2.7, test substance was classified as a sensitizer. The skin sensitization was evaluated through six parameters: body weight, ear thickness, ear weight, lymph node weight, lymph node cell count (LNC), and stimulation index (SI).

(3) $$\mathrm{Stimulation}\;\mathrm{index}\;\left(\mathrm{SI}\right)=\frac{\mathrm{Number}\;\mathrm{of}\;\mathrm{BrdU}-\mathrm{positive}\;\mathrm{LNCs}\;\mathrm{from}\;\mathrm{each}\;\mathrm{mouse}\;\mathrm{exposed}\;\mathrm{to}\;a\;\mathrm{test}\;\mathrm{substance}}{\mathrm{Mean}\;\mathrm{number}\;\mathrm{of}\;\mathrm{BrdU}-\mathrm{positive}\;\mathrm{LNCs}\;\mathrm{in}\;\mathrm{the}\;\mathrm{vehicle}\;\mathrm{control}\;\mathrm{group}}$$

### 2.7. Statistical Analysis

Data were analyzed using the GraphPad Prism 5 (Graph-Pad software ver. 5, San Diego, CA, USA). All data are expressed as the mean ± standard error of the mean (SEM). KeratinoSens™ assay data was compared by one-way ANOVA compared by post hoc Turkey’s pair-wise comparisons. LLNA: BrdU-FCM assay data was compared by unpaired *t*-test. A value of *p* < 0.05 was considered statistically significant.

## 3. Results

### 3.1. Physicochemical Characteristic of NMs

To identify physicochemical properties of silver (Ag)/gold (Au) nanomaterials including AgNMs, PEGylated AgNMs, AuNMs, and PEGylated AuNMs, we analyzed the hydrodynamic size and zeta potential of four NMs (Table 1). The hydrodynamic size of all NMs were increased compared to the primary size when suspended in the working solution. AuNMs and AgNMs showed the largest size in the working solution * (in vitro) and working solution ** (in vivo), respectively. The zeta potential of all NMs were negatively charged using charge of phosphate-buffered saline (PBS) and working solution. All NMs were more positively charged in the medium containing serum than in PBS solution. Further, the NMs to which the functional groups of PEG were attached were slightly more positive than the charge of the original material. According to a LAL test, the endotoxin levels of four NMs were lower than the limit of kit’s detection (0.1 U/mL).

### 3.2. Sensitization Evaluation of NMs in the KeratinoSens™ Assay

**Figure 1:**
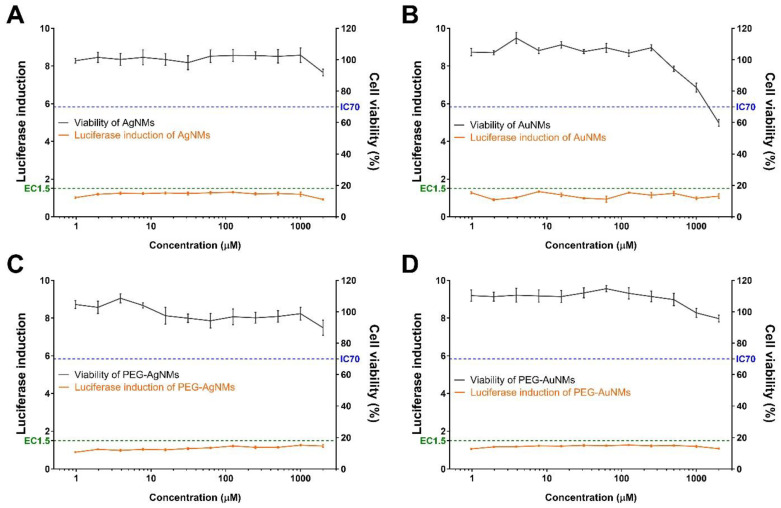
Induction of luciferase activity (orange line) and cell viability (black line) of the KeratinoSens™ assay. The cells were treated with the (**A**) silver nanomaterials (AgNMs), (**B**) gold nanomaterials (AuNMs), and PEGylated nanomaterials for (**C**) AgNMs, and (**D**) AuNMs. Data are expressed as the mean ± SEM (*n* = 6).

**Figure 2:**
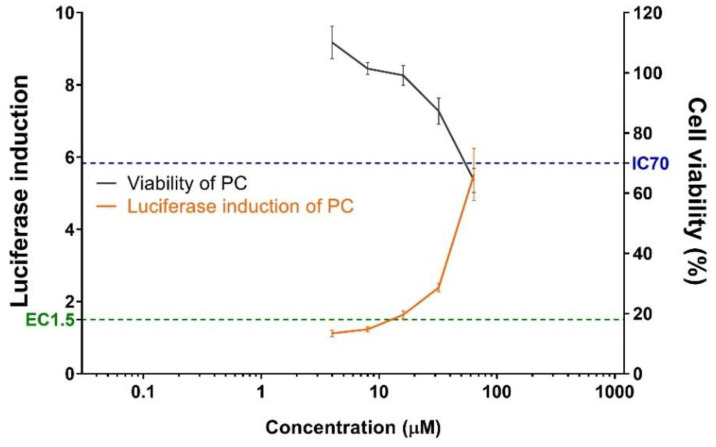
Luciferase activity (orange line) and cell viability (black line) of positive control (trans-cinnamic aldehyde, CASRN. 14371-10-9) in KeratinoSens™ assay. Data are expressed as the mean ± SEM (*n* = 6). The positive control was tested in parallel (concentration: 4–64 µM).3.2. Bronchoalveolar lavage fluid analysis.

The skin sensitization potential of four NMs were validated using the KeratinoSens™ assay (Figure 1). None of the tested NMs (AgNMs, PEG-AgNMs, AuNMs, and PEG-AuNMs) induced the luciferase activity, but the positive control (trans-cinnamic aldehyde) significantly increased the luciferase activity (Figure 2). We also confirmed effect of NMs on cell viability via MTT assays. We showed that AgNMs and PEGylated-NMs treatment did not influence cell viability while the cell viability decreased at high concentration of AuNMs (500–2000 µM) (Figure 1). These data indicate that all tested NMs are not categorized as skin sensitizers. Instead, it could be concluded that these NMs slightly affect the cell viability at high concentrations.

### 3.3. Evaluation of NMs in the LLNA: BrdU-FCM Assay

**Figure 3:**
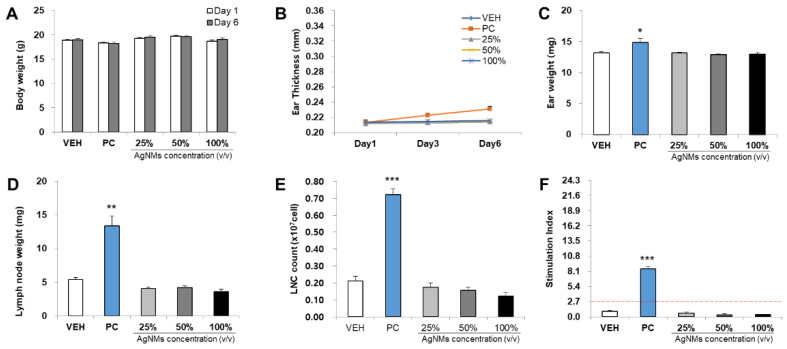
Results of AgNMs skin sensitization potential in LLNA: BrdU-FCM. The assessment parameters were as follows: (**A**) Body weight (g), (**B**) Ear thickness (mm), (**C**) Ear weight (mg), (**D**) Lymph node weight (mg), (**E**) Lymph node cell (LNC) count (×10^7^ cells), and (**F**) Stimulation Index (SI). Data are expressed as the mean ± SEM (*n* = 4). Each treatment group was compared with the vehicle control group to determine the statistical significance. * *p* < 0.05, ** *p* < 0.01, and *** *p* <0.001.

**Figure 4:**
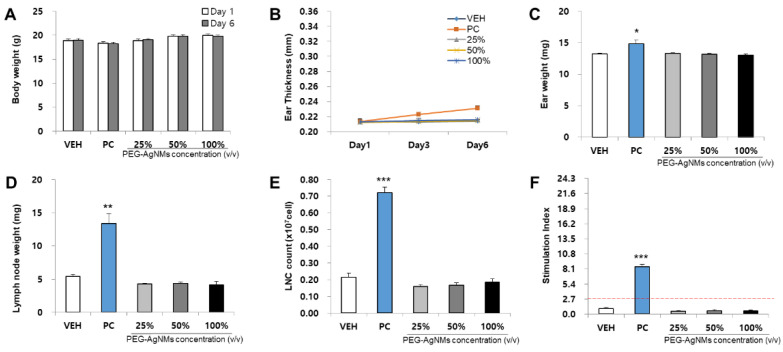
Results of PEG-AgNMs skin sensitization potential in LLNA: BrdU-FCM. The assessment parameters were as follows: (**A**) Body weight (g), (**B**) Ear thickness (mm), (**C**) Ear weight (mg), (**D**) Lymph node weight (mg), (**E**) Lymph node cell (LNC) count (×10^7^ cells), and (**F**) Stimulation Index (SI). Data are expressed as the mean ± SEM (*n* = 4). Each treatment group was compared with the vehicle control group to determine the statistical significance. * *p* < 0.05, ** *p* < 0.01, and *** *p* <0.001.

**Figure 5:**
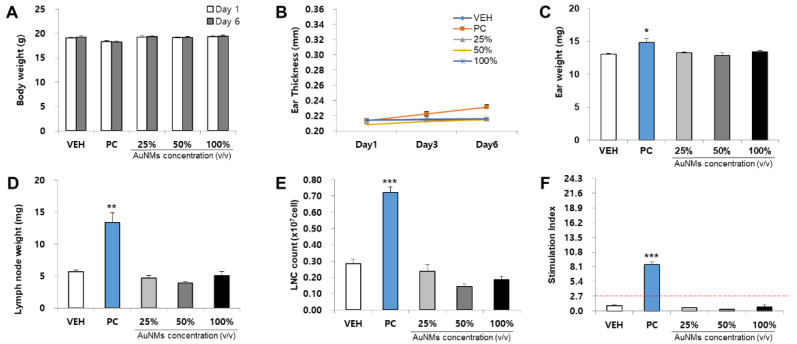
Results of AuNMs skin sensitization potential in LLNA: BrdU-FCM. The assessment parameters were as follows: (**A**) Body weight (g), (**B**) Ear thickness (mm), (**C**) Ear weight (mg), (**D**) Lymph node weight (mg), (**E**) Lymph node cell (LNC) count (×10^7^ cells), and (**F**) Stimulation Index (SI). Data are expressed as the mean ± SEM (*n* = 4). Each treatment group was compared with the vehicle control group to determine the statistical significance. * *p* < 0.05, ** *p* < 0.01, and *** *p* <0.001.

**Figure 6:**
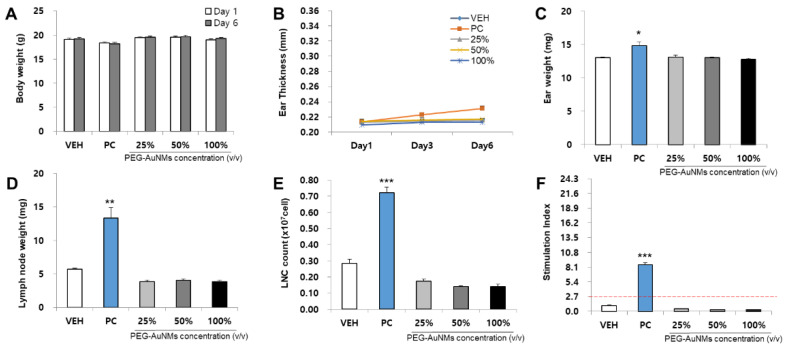
Results of PEG-AuNMs skin sensitization potential in LLNA: BrdU-FCM. The assessment parameters were as follows: (**A**) Body weight (g), (**B**) Ear thickness (mm), (**C**) Ear weight (mg), (**D**) Lymph node weight (mg), (**E**) Lymph node cell (LNC) count (×10^7^ cells), and (**F**) Stimulation Index (SI). Data are expressed as the mean ± SEM (*n* = 4). Each treatment group was compared with the vehicle control group to determine the statistical significance. * *p* < 0.05, ** *p* < 0.01, and *** *p* <0.001.

The skin sensitization potential of four NMs was analyzed using the LLNA: BrdU-FCM assay (Figure 3, Figure 4, Figure 5 and Figure 6). As a result of body weight measurement, no significant increase/decrease was observed in any test NMs. Regarding ear thickness, a significant increase was observed on day 6 in the positive control group compared with other groups. Ear weight, lymph node weight, lymph node cell count (LNC), and stimulation index (SI) were also significantly increased in the positive control group. Significant results were not found, and the SI value was lower than 2.7 in all test NMs. Consequently, all test NMs were judged as non-sensitizing according to the test guideline 442B criteria.

## 4. Discussion

PEG is a polyether compound used in various fields from industrial manufacturing to medicine. Due to its low toxic and highly soluble properties, PEG has been applied in pharmaceutical and biomedical applications. PEG can bind to various target molecules such as peptides, proteins, or oligonucleotides and act as a drug mediator to extend their residence in the body [20]. By coating PEGs on the surface of nanoparticles, they can be used to enhance systematic drug delivery. PEG coatings would be an important factor in the development phase of NPs for drug and gene delivery applications. Ongoing studies are needed on how the properties of PEG coatings affect the biodistribution and clearance of NPs. PEG is also well known for its use as a binding and dispersing agent, as it can help to separate the particles and to prevent agglomeration [2].

However, one recent study reported that PEG causes side effects such as anaphylaxis by type I hypersensitivity mechanism [21,22]. Therefore, there is a need to identify the toxicity of the PEG precisely. In this paper, we performed the TG 442D in vitro test, which is the second key event of the OECD skin sensitization test method and the TG 442B in vivo test, which is the fourth key event. The aim of this study was to evaluate the skin sensitization properties of nanoparticles with or without PEG.

Nanomaterials can permeate the skin in conditions such as small size, easily released ions, or damaged skin. Gold/silver NMs are known to have the potential to be absorbed into intact skin due to their small size [23]. Additionally, they are described as low hazard compared to other NPs and are extensively used in fields such as surgical and handling tools, food, clothing, cosmetics, disinfectants, and wound dressings. [24]. For this reason, in this study, gold/silver NMs, which are known to have absorption potential into the skin, were used to evaluate the immunogenicity and skin sensitization with or without PEG [25].

According to the cell viability results, there was a difference between the cell viability of AuNMs and that of AuNMs-PEG at high concentrations (Figure 1B,D). While AuNPs significantly reduced cell viability at high concentrations (500–2000 μM), the effect of PEGylated-AuNMs was relatively slight under the same conditions. Consistent with our results, there are other reports that suggest AuNPs downregulate the cell viability, depending on dosage [25,26,27]. The most well-known mechanism of AuNPs-induced cytotoxicity is the oxidative stress due to reactive oxygen species (ROS), and overproduction of ROS is known to induce apoptosis [25]. In addition, a recent study showed that high concentration of AuNPs inhibits mitochondria functioning via diminishing the mitochondria membrane potential, resulting in apoptosis [25].

There are some studies indicating that PEG repairs the plasma membrane and reduces the oxidative stress in a spinal cord injury model [28]. When an injury model of the isolated spinal cord was treated with PEG, it showed the effect of recovering the damaged membrane [29]. PEG reduces not only membrane damage, but also the generation of ROS and apoptosis. Therefore, these findings suggest that PEG’s protective effect could possibly be applied to other types of cells [29]. Intriguingly, our results showed that PEG-coated AuNMs were less cytotoxic compared to AuNMs (Figure 1B,D). These results indicate that PEG might be related to inhibition of ROS production and apoptosis, although further experiments are needed.

As a result of the in vivo study, all test substances were determined to be non-sensitizing, which could be interpreted in two aspects. First, gold/silver nanomaterials may agglomerate in working solutions and may not be absorbed from intact skin due to an increase in size [30]. It has been verified that the size of nanoparticles is an important factor in the inflammatory response [31]. When not absorbed, nanomaterials that cause sensitization when exposed to the body can be defined as non-sensitizing materials. Further studies are needed using a model that can be absorbed into the skin such as damaged skin. Then, the toxicity of nanomaterials in the body could be identified [32,33]. Secondly, it can be judged that PEG is a non-sensitization substance. As gold/silver NMs are recognized as external substances by immune cells, they can induce an inflammatory response including activation of macrophages, neutrophils, and helper T cells [34,35]. As a result, it could lead to the expression of cytokines such as TNF-α, IL-1β, IL-6, IL-12, and IL-18. In addition, it has been reported that AgNMs can be classified as weak skin sensitizers [36]. However, in our study, sensitization was not induced in a total of four nanomaterials including PEG functional groups. Moreover, the previous study regarding the covalent attachment of PEG to proteins reported that PEG coatings decreased the immunogenicity of proteins [2].

In the present study, our results only dealt with the effects of PEGylation on two types of nanomaterials. As commercially available PEGylated nanomaterials can be manufactured according to various cores, it is necessary to secure various toxicological data through additional research in order to obtain the safety of PEGylated nanomaterials. Furthermore, it is needed to establish a nanomaterial-specific test guideline for laying the foundation for accurate risk assessment and to establish regulations and policies for protecting the production workers and subjects directly involved in exposure to the nanomaterials.

## 5. Conclusions

In this study, evaluation of skin sensitization by four NMs showed the non-sensitization with or without PEG functional groups through two skin sensitization test methods. In addition, we confirmed significant differences in cytotoxicity within NMs with or without PEG. Furthermore, this study found that PEGylation of gold nanomaterials could induce a slight decrease in cytotoxicity upon the keratinocyte exposure. Further experiments are required to identify the major role of PEG in cytotoxicity.

## Figures and Tables

**Table 1:** Physicochemical properties of tested nanomaterials in in vitro and in vivo assay.

Characteristic	AgNMs	PEG-AgNMs	AuNMs	PEG-AuNMs
Product NO. info	730785 (Sigma)	796301 (Sigma)	753610 (Sigma)	NCXAUXU30 (Sigma)
Primary size (nm)	10	40	20	30
Hydrodynamic size (nm) ^†^				
PBS	51.4 ± 20.1	60.1 ± 3.1	31.0 ± 2.0	35.9 ± 1.0
Working solution *	17.6 ± 1.5	65.7 ± 1.7	183.9 ± 16.8	41.2 ± 0.7
Working solution **	145.0 ± 5.9	124.4 ± 2.5	117.9 ± 2.3	99.8 ± 2.3
Polydispersity (PDI) ^††^				
PBS	0.22 ± 0.05	0.09 ± 0.01	0.38 ± 0.02	0.09 ± 0.01
Working solution *	0.40 ± 0.06	0.25 ± 0.00	0.21 ± 0.05	0.30 ± 0.03
Working solution **	0.44 ± 0.06	0.18 ± 0.03	0.35 ± 0.04	0.20 ± 0.03
Zeta potential (mV) ^†††^				
PBS	−47.2 ± 1.4	−13.5 ± 0.6	−39.3 ± 1.1	−40.8 ± 1.5
Working solution *	−36.3 ± 3.8	−9.9 ± 1.2	−21.9 ± 6.2	−15.6 ± 1.5
Working solution **	−11.5 ± 0.6	−9.4 ± 0.9	−10.6 ± 0.7	−12.2 ± 0.7
Endotoxin (EU/mL)	<0.1	<0.1	<0.1	<0.1

^†^ Hydrodynamic sizes of each nanomaterial in the following solutions (PBS, working solution) are displayed. * Working solution (KeratinoSens™ assay) was composed of PBS stock (1%) + DMEM, containing 1% FBS. ** Working solution (LLNA: BrdU-FCM assay) composed of DW stock (10%) + DMF, containing 3% mouse serum. ^††^ Polydisperity of each nanomaterial in the following solutions (PBS, working solution) is displayed. ^†††^ Zeta potential of each nanomaterial in the following solutions (PBS, working solution) is presented. Data are expressed as mean ± SEM, *n* = 6; NMs = nanomaterials, PBS = phosphate-buffered saline, EU = endotoxin unit, DMEM = Dulbecco’s Modified Eagle’s Medium, FBS = Fetal bovine serum, DW = distilled water, and DMF = N,N-Dimethylformamide.

## Data Availability

The original contributions presented in the study are included in the article further inquiries can be directed to the corresponding authors.
